# Patient-reported treatment outcomes and safety of direct-to-consumer teledermatology for finasteride treatment in male androgenetic alopecia: A cross-sectional study

**DOI:** 10.1177/20552076231205740

**Published:** 2023-10-04

**Authors:** Johannes von Büren, Inga Hansen, Julian Kött, Florian Schröder, Juliana Veneroso, Stefan W. Schneider, Finn Abeck

**Affiliations:** 1 Wellster Healthtech Group, Munich, Germany; 2 Department of Dermatology and Venereology, 37734 University Medical Center Hamburg-Eppendorf, Hamburg, Germany

**Keywords:** Direct-to-consumer, teledermatology, telemedicine, androgenetic alopecia, hair loss, finasteride

## Abstract

**Objective:**

The use of direct-to-consumer (DTC) teledermatology platforms has increased, particularly for androgenetic alopecia (AGA). However, little is known about the efficacy and safety of these platforms. This study aimed to investigate the patient-reported treatment outcomes and safety of DTC teledermatology for the finasteride treatment of male AGA.

**Methods:**

This retrospective, cross-sectional study used data from a German DTC platform for finasteride treatment between December 2021 and January 2023. Patient-reported outcomes were collected through voluntary follow-up questionnaires provided to the patients six weeks after the first prescription to assess treatment outcomes and safety.

**Results:**

Data collection included 2269 patients. Of all patients who answered the follow-up questionnaire (*n* = 191), 79% (150 out of 191) self-reported positive changes in hair appearance, and 59% (113 out of 191) reported an improvement in self-esteem under treatment. Patients with self-reported positive changes in hair appearance were more likely to report improved self-esteem (*P* < 0.0001). Treatment-related adverse events occurred in 12% (22 out of 191) of the patients. Full treatment adherence was reported in 87% (167 out of 191) of patients.

**Conclusion:**

From the patient's perspective, DTC teledermatology has the potential to improve hair appearance and self-esteem. Our results suggest that it may be an effective and safe treatment option for men with AGA, justifying low-threshold access. However, treatment-related adverse events should be closely monitored during follow-up. Further studies are required to evaluate the long-term effects of the DTC teledermatology treatment. By collecting real-world data, teledermatology platforms could be useful beyond their primary focus and could play an important role in the context of future research.

## Introduction

Direct-to-consumer (DTC) teledermatology has changed how patients receive dermatological care. Using these services, patients can receive dermatology consultations, including individual treatment plans and prescription medications without an in-person visit. The benefits of DTC teledermatology include facilitated access to care and a high level of patient convenience.^1^

The coronavirus pandemic has fundamentally influenced healthcare delivery. Despite the social isolation during the pandemic, teledermatology proved to be a powerful tool for care delivery.^2^ Even after the end of the pandemic, it has shown the potential to play an important role in the current and future care of patients.^3^ According to a recent study, most dermatologists have a good knowledge of telemedicine and support its implementation. In addition to reducing costs and waiting times, teledermatology offers the potential benefit of reducing the frequency of patient visits to clinics. However, dermatologists are also aware of the challenges that these technologies can bring, such as diagnostic accuracy, data protection, and additional administrative obligations.^4^

Different types of DTC platforms can be distinguished: while some platforms offer medical advice for all skin disorders using photo diagnosis, others offer targeted medical care for patients with a selected disease.^1^ In particular, the prevalence of digital health platforms offering telemedicine diagnosis and treatment for hair loss has increased.^5^ With up to 80% of men being affected by androgenetic alopecia (AGA) during their lifetime, it is the most common hair loss disorder worldwide.^6^ Finasteride is its most effective therapy in men.^6^ Significant limitations in the quality of life may be associated with the disease.^7,8^ In addition, studies have shown decreased self-esteem in patients with AGA.^9^ Previous studies provided information on the patient population on DTC teledermatology platforms for men with AGA.^10,11^ The main reasons for using teledermatology to treat hair loss are convenience and discretion.^10^ The quality of treatment is considered equivalent to that of outpatient dermatology consultation by most patients.^11^ However, as the number of patients using these platforms is growing rapidly, further studies on this type of medical care, particularly with regard to its efficacy and patient safety, are critical.^10^ Patients’ perspectives in health research have become increasingly important in recent years. They can be evaluated by using patient-reported outcomes (PRO) reported directly by the patient, with no evaluation of the patient's response by a clinician or anyone else.^12^ PROs were also recommended as essential components for evaluating telemedicine services.^13^ This study aimed to investigate the potential of teledermatology for the treatment of male AGA by analyzing PRO regarding telemedical treatment outcomes and safety in patients from a DTC platform for finasteride treatment available in Germany.

## Materials and methods

### Study design

Data for this retrospective cross-sectional study were provided by Wellster Healthtech Group, the provider of “www.myspring.com,” a DTC teledermatology platform available in Germany for male AGA. This platform is comparable to DTC platforms in the USA, such as Keeps, Hims, and Roman.^5^ Patient data were collected via structured questionnaires between 1 December 2021 and 31 January 2023. As part of the telemedicine treatment, patients were asked about AGA characteristics, finasteride contraindications, and possible medication interactions. AGA was classified using the Hamilton-Norwood scale. Patients can select the stage that most closely describes their current hair state based on the images. Since stage I of the Hamilton-Norwood scale indicates no visible hair loss, images from grades II to VII were selected. In addition, it was possible to upload a photograph of the current hair status. A video consultation was also available for an additional fee. Male patients aged 18 years or older and with self-assessed AGA were eligible for the study. AGA diagnosis was confirmed by a physician based on the patients’ statements. Prescriptions were only issued to patients without contraindications. Following the prescription, finasteride was ordered from a cooperative online pharmacy. Information regarding its efficacy and the occurrence of possible treatment-related adverse events was provided via email as a standard procedure to ensure patient safety. In addition to finasteride, over-the-counter products (topical minoxidil, biotin tablets, biochanin A serum, and baicapil shampoo [a combination of the plants scutellaria baicalensis, soy, and wheat sprouts]) were also available for purchase through the DTC platform. Subsequently, further data regarding PROs (treatment efficacy, self-esteem changes, treatment-related adverse events, and adherence) and concomitant medication were obtained using follow-up questionnaires sent by email six weeks after the first prescription. A list of 27 treatment-related adverse events was presented to patients to identify possible side effects. Free-text comments were also possible. The follow-up questionnaire was voluntarily administered. Only questionnaires completed by at least 90% of the participants were included in the analysis.

### Statistical analysis

Data analyses were performed using GraphPad Prism software, version 8 (GraphPad Software, San Diego, CA, USA). Descriptive statistics were summarized as mean ± standard deviation. The Mann-Whitney *U* test was used to compare differences in the mean age. The chi-square test was used to compare the AGA stages and age groups. All statistical tests were two-sided, and the level was set at 5.0% (*p* ≤ 0.05). Data were presented as mean ± standard deviation.

## Results

Data of 2269 patients who received finasteride treatment on the DTC platform between 1 December 2021 and 31 January 2023 were included in the analysis (P1). In the P1 group, the mean age was 32 ± 8.3 years (range 19–84), while 33% (745 out of 2269) of the patients were aged between 25 and 30 years. A total of 48% (1079 out of 2269) of the patients were classified as grade III according to the Hamilton-Norwood scale based on patient self-assessment (Table 1).

**Table 1.** General characteristics of patients from a German online prescription platform for AGA.

	All finasteride prescriptions (P1)	Follow-up survey (P2)	*P* value*
	*n* = 2269	*n* = 191	
Age			0.081
Mean **±** SD	32 ± 8.3	33 ± 9.2	
Range	19–84	19–64	
Age group, *n* (%)	2269 (100)	191 (100)	0.33
20–24	393 (17)	33 (17)	
25–30	745 (33)	51 (27)	
31–35	491 (22)	41 (22)	
36–40	329 (15)	33 (17)	
≥ 41	311 (18)	33 (17)	
Norwood-Hamilton scale, *n* (%)	2269 (100.0)	191 (100.0)	0.14
Grade II	374 (17)	20 (11)	
Grade III	1079 (48)	100 (52)	
Grade IV	563 (25)	47 (25)	
Grade V	205 (9.0)	16 (8.4)	
Grade VI	36 (1.6)	6 (3.1)	
Grade VII	12 (0.5)	2 (1.0)	

*n*: number of patients; SD: standard deviation; AGA: androgenetic alopecia.

Further analysis was conducted on a subset of 8.4% (191 out of 2269) of all patients (P2) who completed the follow-up questionnaire six weeks after the first finasteride prescription. The P2 group did not differ significantly from the P1 group in terms of age, age group, or distribution according to the Hamilton-Norwood scale (Table 1).

**Figure 1.**
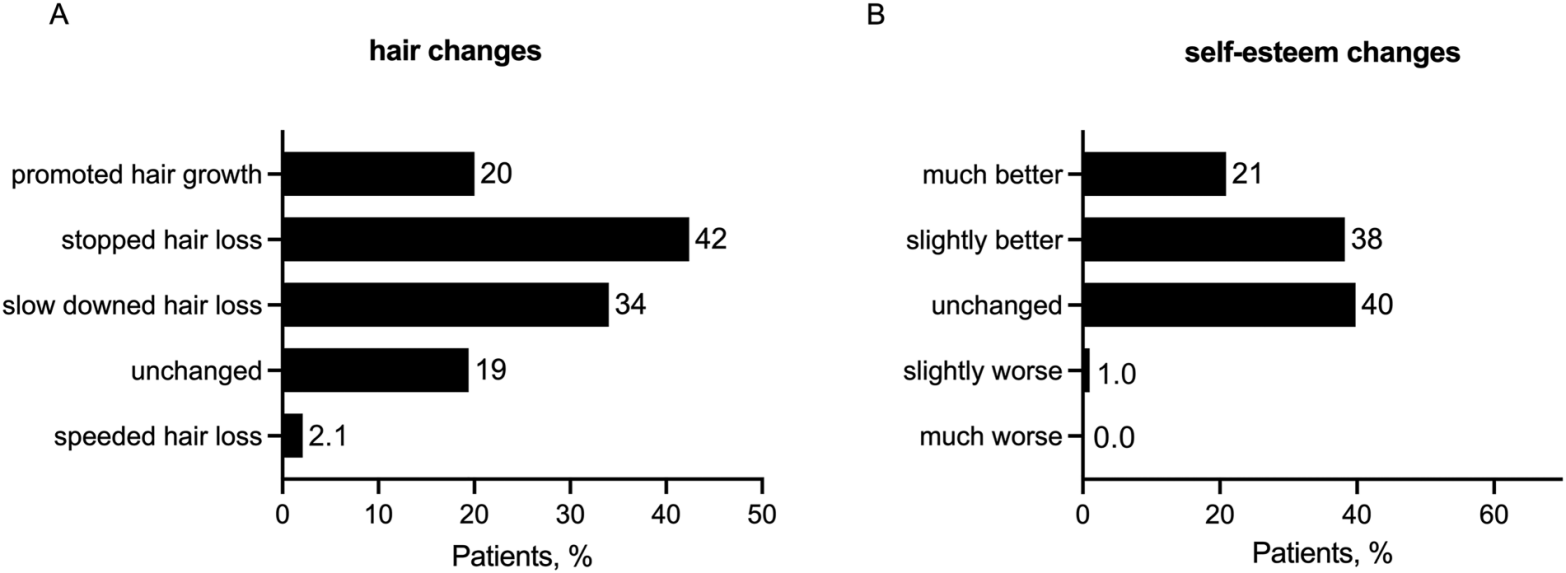
Patient self-reported treatment outcomes from the follow-up questionnaire six weeks after the first finasteride prescription (*n* = 191). (A) Patients were asked how their hair appearance has changed with therapy (multiple responses possible). (B) Patients were asked how their self-esteem has changed with therapy.

In 79% (150 out of 191) of the patients, positive changes in hair appearance were self-reported, whereas, in 19% (37 out of 191), no change in hair appearance has been recognized to date (Figure 1A, multiple answers possible). No differences in hair changes under treatment were found when comparing patients in the self-reported early stages to those in the late stages of AGA according to the Norwood-Hamilton scale (Fisher's exact test, *P* = 0.20). Of all respondents, 59% (113 out of 191) reported an improvement in self-esteem after telemedical treatment (Figure 1B). Patients with self-reported positive changes in hair status (70% vs. 20%, Fisher's exact test, *P* < 0.0001) and those with self-reported early-stage AGA (Norwood-Hamilton scale grades II and III; 66% vs. 48%, Fisher's exact test, *P* = 0.022) were more likely to report self-esteem improvement under therapy.

A total of 51% (97 out of 191) of the patients reported using products for hair loss treatment in addition to finasteride. Minoxidil (33%, 63 out of 191) was mentioned most frequently followed by biotin (17%, 32 out of 191). In addition, 25% (24 out of 97) of patients receiving co-medication reported using at least two products besides finasteride. One patient was identified with the concomitant use of topical finasteride, which is contraindicated. Patients receiving concomitant AGA treatment were more likely to self-report positive effects on hair appearance during therapy (77% vs. 60%, Fisher's exact test, *P* = 0.012).

**Figure 2.**
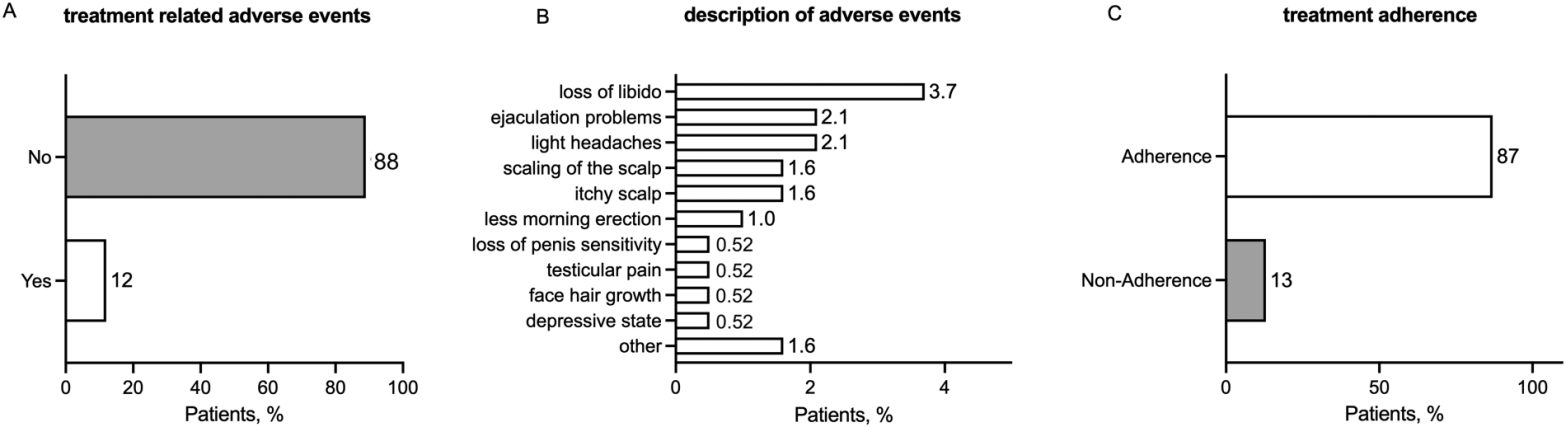
Patient self-reported data from the follow-up questionnaire six weeks after the first finasteride prescription (n = 191). (A) Patients were asked if treatment-related adverse events occurred with the therapy. (B) Patients who experienced adverse events were asked for detailed information about the adverse events. (C) Patients were asked if they used finasteride exactly as recommended by the physician (once daily).

A total of 12% (22 out of 191) of patients reported treatment-related adverse events (Figure 2A), with loss of libido being the most cited (3.7%, 7 out of 191), followed by ejaculation problems (2.1%, 4 out of 191), and light headaches (2.1%, 4 out of 191) (Figure 2B). Patients receiving concomitant AGA treatment reported significantly higher rates of treatment-related adverse events (17% vs. 6.4%, Fisher's exact test, *P* = 0.040). According to the patients’ feedback, 87% (167 out of 191) took finasteride exactly as recommended by the telemedical physician (once daily) (Figure 2C). All non-compliant patients reported taking finasteride at lower doses or less frequently than recommended. None of the patients reported finasteride overdose.

## Discussion

To the best of our knowledge, this is the first study to evaluate the self-reported treatment outcomes and safety of telemedicine in men with AGA receiving finasteride. The inclusion of PROs in research and clinical practice allowed a more complete understanding of the impact of the therapy on patients.^12^ This valuable information is increasingly being used in telemedicine to study this relatively new form of care delivery.^13^

In our study, eight out of 10 self-reported a positive change in hair appearance as early as six weeks, indicating the efficacy of finasteride in the treatment of male AGA. Patients on concomitant AGA treatment products were more likely to self-report an improvement in hair appearance than those without. One in two patients reported the use of other products in addition to finasteride, with topical minoxidil being the most common. Combination therapy with oral finasteride and topical minoxidil has been shown to be superior to monotherapy and may be used according to the current guidelines, particularly in motivated patients, to improve treatment efficacy.^7,14^ The high proportion of patients using multiple hair loss products indicates a high willingness to incur treatment effort and costs. This indicates a high disease burden, which has been described in detail for AGA.^7,8^ There are no valid scientific data on the benefits of biotin therapy in patients with hair loss, especially in patients with normal biotin levels.^15^ DTC platforms have been criticized for selling non-evidence-based therapies such as biotin.^16^ In our study population, only 17% of the finasteride patients were taking biotin. This rather low proportion suggests that the focus of DTC treatment is on the use of evidence-based treatments such as finasteride and minoxidil.

In addition to physical parameters, positive psychological effects were reported. Most patients reported improved self-esteem after the telemedicine AGA treatment. The initial positive effects of finasteride on hair loss may explain this observation, as patients with self-reported positive changes in hair status are more likely to report self-esteem improvements under therapy. A study of 27 male patients with AGA has shown that quality of life improves with finasteride therapy.^17^ Interestingly, in the same study, there was no difference in the quality of life between patients with high and low treatment responses. The authors concluded that subjective satisfaction with the treatment response does not always correlate with objective parameters.^17^

Our study is the first to demonstrate that self-esteem in men with AGA may be improved by DTC finasteride treatment. The improvement in self-esteem may also be related to the fact that patients start to actively manage the disease as well as the education about hair loss and treatment options being provided by the DTC platform. The potential of teledermatology to have a positive impact on patients’ quality of life is demonstrated by the finding that self-esteem improved after only six weeks of therapy, even though only incipient hair changes can be expected during this period. In addition, telemedicine improves access to care. According to our previous study, most patients did not receive finasteride therapy or visit a doctor for hair loss before starting teledermatology treatment.^11^

The patient-reported treatment outcomes “treatment efficacy” and “self-esteem changes” examined in our study suggest that teledermatology can be an effective treatment option for men with AGA.

Off-label prescriptions are commonly used in the field of dermatology.^18^ A shortcoming of DTC platforms, as described in the literature, is that patients can only be offered well-known treatment options and may be denied off-label treatments.^16^ Currently, DTC teledermatology cannot provide the same level of personalized care as traditional in-person visits. However, from our perspective, DTC platforms offer the great advantage of providing insights into the side-effect profile of prescribed drugs using standardized medical questionnaires for follow-up and patient monitoring. This also enables the long-term care of patients in telemedicine, which is often criticized as a shortcoming of DTC platforms.^16^

In our study, most patients reported no treatment-related adverse events. The most common adverse events reported were sexual side effects such as decreased libido and problems with ejaculation. These side effects are compatible with the known side effects of finasteride therapy.^19^ Other reported side effects, such as scalp itching or scaling, are not typical of finasteride and are most likely caused by the concurrent minoxidil treatment. One patient in the follow-up group was concurrently treated with topical finasteride in addition to oral finasteride, which is contraindicated. DTC platforms offer the advantage of automatic mechanisms that prevent contraindicated medications from being ordered simultaneously. This could significantly improve patient safety. However, neither in the outpatient setting nor in telemedicine can it be completely avoided that patients find ways to combine medications that are contraindicated in this application. The patient in our study was informed of the contraindicated combination therapy.

Despite the low incidence of adverse events, efforts to ensure patient safety are crucial. Digital health platforms should collaborate with office-based physicians to enhance the benefits and safety of DTC teledermatology. In case of serious adverse events, patients can be referred directly to local contacts for medical support.^20^ Synergizing DTC teledermatology and in-person care is an interesting model that may play an important role in the future.^21^

As telemedicine continues to gain importance in healthcare, ethical, social, and political aspects must also be considered. Data information and availability are provided here as examples. With the help of electronic patient records, patient information can be rapidly made available to the treating physician, simplifying everyday treatment. Simultaneously, there is a need to identify methods to protect the security and integrity of patient data.^22^ In addition, DTC platforms can be useful beyond their primary focus by collecting real-world digital data. This valuable information can be used in the future to investigate various research questions to improve patient care.

Full treatment adherence was reported in 87% of the patients. To date, there are no data on treatment adherence in patients taking finasteride. Our data showed a high level of adherence among patients with hair loss treated with telemedicine, which is essential for safe and effective treatment. Similarly, high adherence has been reported in patients using a DTC platform to diagnose and manage skin diseases.^23^

The PRO “treatment-related adverse events” and “treatment adherence” examined in our study provided evidence that DTC teledermatology is a safe treatment option for men with AGA.

The limitations of this study include its retrospective cross-sectional design, from which no causal statements can be derived. The response rate to the follow-up questionnaire was 8.4%, indicating that a selection bias could not be excluded. When asked about treatment-related adverse events, patients with side effects might be more likely to participate than those without side effects. However, the general characteristics of the 191 patients in the follow-up group (P2) were comparable to those of the 2269 patients who were treated on the platform (P1). Because the follow-up questionnaires were sent to patients six weeks after the initial finasteride prescription, our study analyzed the short-term self-reported treatment effectiveness and safety of telemedicine for the treatment of AGA in men. As the full efficacy of finasteride therapy can only be assessed after approximately three to six months of consistent use, further studies with longer observation periods are needed. Potential confounding factors, such as the concomitant use of minoxidil, may have affected the PRO studied. The duration of concomitant use at the time of the survey is also unknown. Furthermore, the study group definition was based on the patients’ self-assessment of AGA. The use of objective measures, such as trichoscopy, to confirm the diagnosis or assess treatment response is not possible in a pure telemedicine setting.^16^ However, in AGA treatment, subjective patient satisfaction with the treatment response is very important and should be considered when deciding whether to continue or discontinue finasteride therapy.^17^

## Conclusion

Patients with AGA reported high levels of subjective distress. The use of DTC teledermatology for finasteride treatment of male AGA has the potential to improve patient self-reported treatment outcomes. Side effects associated with DTC finasteride treatment may occur but affect only a small proportion of patients. Additionally, the patients showed a high level of treatment adherence. The results of our study suggest that DTC teledermatology is an effective and safe treatment option for men with AGA. Therefore, low-threshold access to DTC finasteride for male patients with AGA can be justified if treatment-related adverse events are closely monitored with adequate patient follow-up. However, further studies are required to evaluate the long-term effects of DTC teledermatology in male patients with AGA. In addition, the potential shortcomings of DTC platforms, such as the distribution of non-evidence-based products and the limitations of individualized treatment, must be considered. By collecting real-world data, these platforms could be useful beyond their primary focus and could play an important role in the context of future research.
